# Low-level variant calling for non-matched samples using a position-based and nucleotide-specific approach

**DOI:** 10.1186/s12859-021-04090-y

**Published:** 2021-04-08

**Authors:** Jeffrey N. Dudley, Celine S. Hong, Marwan A. Hawari, Jasmine Shwetar, Julie C. Sapp, Justin Lack, Henoke Shiferaw, NISC Comparative Sequencing Program, Jennifer J. Johnston, Leslie G. Biesecker

**Affiliations:** 1 National Human Genome Research Institute, National Institutes of Health, 50 South Drive Room 5140, Bethesda, MD 20892 USA; 2 NIAID Collaborative Bioinformatics Resource, National Institutes of Allergy and Infectious Diseases, National Institutes of Health, Bethesda, MD USA; 3 Advanced Biomedical Computational Science, Frederick National Laboratory for Cancer Research, Frederick, MD USA; 4 NIH Intramural Sequencing Center, National Human Genome Research Institute, National Institutes of Health, Rockville, MD, USA

**Keywords:** Mosaic variants, Prediction of mosaic variants, Somatic overgrowth disorder

## Abstract

**Background:**

The widespread use of next-generation sequencing has identified an important role for somatic mosaicism in many diseases. However, detecting low-level mosaic variants from next-generation sequencing data remains challenging.

**Results:**

Here, we present a method for Position-Based Variant Identification (PBVI) that uses empirically-derived distributions of alternate nucleotides from a control dataset. We modeled this approach on 11 segmental overgrowth genes. We show that this method improves detection of single nucleotide mosaic variants of 0.01–0.05 variant allele fraction compared to other low-level variant callers. At depths of 600 × and 1200 ×, we observed > 85% and > 95% sensitivity, respectively. In a cohort of 26 individuals with somatic overgrowth disorders PBVI showed improved signal to noise, identifying pathogenic variants in 17 individuals.

**Conclusion:**

PBVI can facilitate identification of low-level mosaic variants thus increasing the utility of next-generation sequencing data for research and diagnostic purposes.

**Supplementary Information:**

The online version contains supplementary material available at 10.1186/s12859-021-04090-y.

## Background

Next-generation sequencing (NGS) has greatly advanced our ability to investigate the biological consequence of somatic mosaic variants. In cancer, detection thresholds for mosaic variants are often set around 0.10 variant allele fraction (VAF) on the basis that a tumor with a clone at less than 0.20 cellular contribution is not an appealing therapeutic target. In non-malignant mosaic overgrowth, VAF may be well below 0.10 and may not correlate with apparent affection status of the sampled tissue [1, 2]. Furthermore, affected tissue (e.g., brain) may not be accessible for sampling in which case a proxy tissue (peripheral blood or skin) may be used as a sample source with a VAF well below 0.05 [3]. Identifying somatic mosaic variants at low VAF is challenging because of the sequencing error that is inherent to the technology [4, 5]. Current somatic mosaic variant callers address low-level detection with a variety of approaches and target applications [6]. However, maximizing sensitivity while reducing false positives near the limits of sequencing error is an ongoing challenge.

Most somatic mosaic variant callers employ a matched sample calling strategy, likely due to their development for applications in cancer diagnostics [7] and research [8]. In this strategy, sequence data from affected (tumor) and unaffected (non-tumor) tissues are compared to identify variants with VAF that are significantly higher in the tumor sample. In mosaic overgrowth disorder testing the inability to unambiguously identify unaffected tissues, combined with low VAF in some affected tissues, requires a different approach to variant calling. Furthermore, an evaluation of current unmatched callers has shown that most struggle to maintain both high sensitivity and precision at VAF < 0.05 [9]. Consequently, developing appropriate methods for detecting low-level (VAF < 0.05) variants in unmatched samples is critical to interpretation of NGS data in these conditions.

In this work we present a simple and highly sensitive method designated as Position-Based Variant Identification (PBVI) that enables sensitive low-level variant calling by modeling NGS error directly from a control dataset. PBVI focuses on single-nucleotide variants (SNVs) since they account for a majority of currently-identified pathogenic variants in individuals with somatic overgrowth disorders. The underlying assumption of PBVI is that the empirical distribution of alternate nucleotides in NGS data from a control dataset can be used to determine the probability that a candidate variant with a certain VAF is not sequencing error. Previous work has shown clear patterns in NGS error on Illumina platforms due to factors including; preferential nucleotide misincorporation for a given reference nucleotide, sequence mappability, variant position within a read, and the tendency of certain genomic positions to be error-prone due to the preceding sequence of nucleotides. While previous studies have shown that modeling empirical distributions of nucleotides in control data can be used to aid identification of high-confidence variants, PBVI builds on previous work by assessing forward and reverse strands independently allowing for preceding sequence to be considered in our variant calling algorithm with simplified statistical approach [10–15].

We test the performance of PBVI on simulated data and compare the performance to two mosaic variant callers, VarDict [16] and LoFreq [17]. We validate PBVI by detecting variants in a set of samples from 26 individuals with somatic overgrowth disorders again comparing the performance of this method to VarDict and LoFreq.

## Results

### Robustness of direct modeling approach

**Fig. 1:**
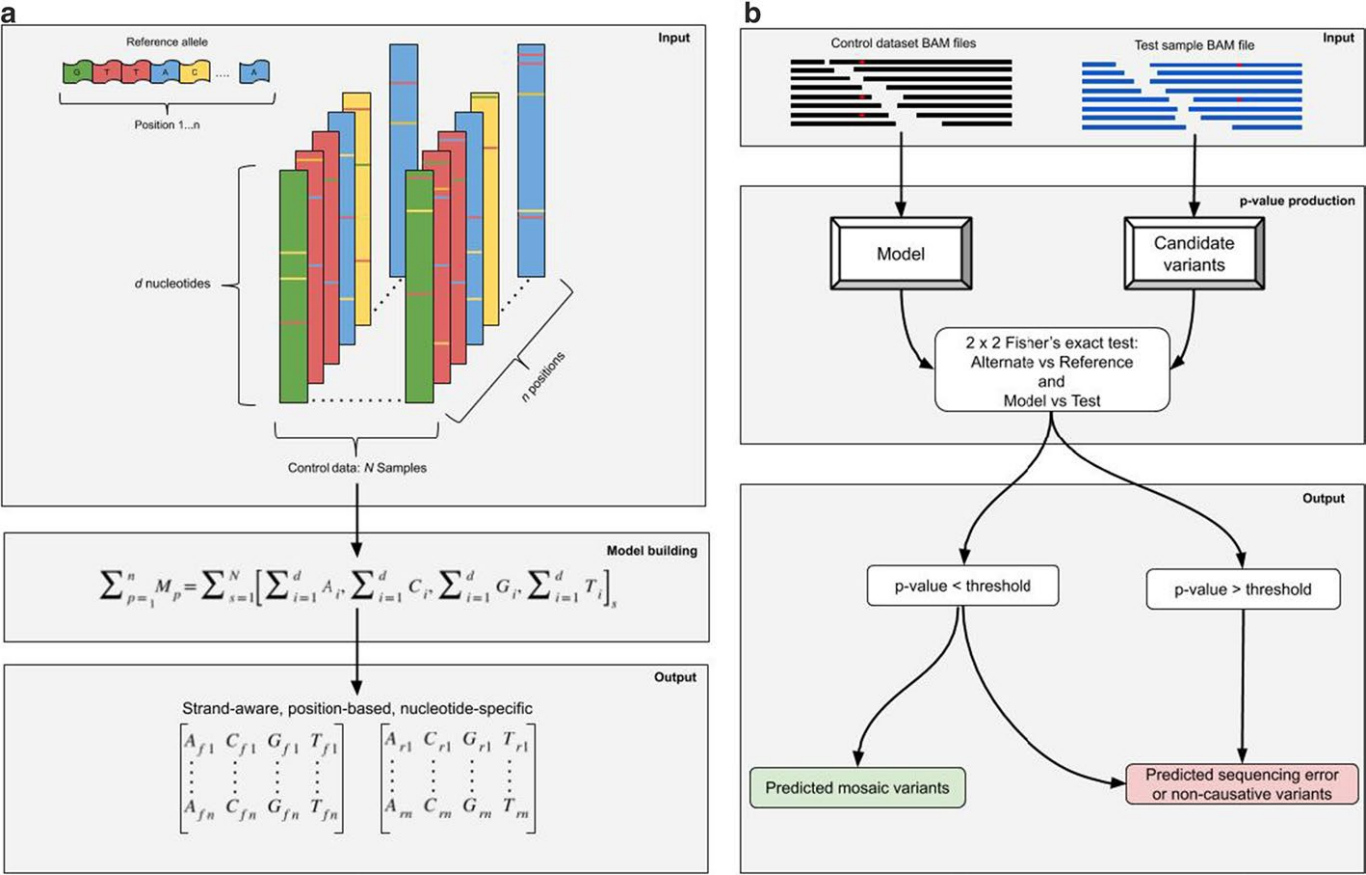
Overview of approach. **a** Model building from a control dataset. Nucleotide counts from each BAM file in the control dataset were used to build the model. Nucleotides (*d*) are summed at each position (*n)* and aggregated across all BAM files in the control dataset (*N*). Position-Based Variant Identification (PBVI) splits counts into two sperate matrices dependent on whether the count is on the forward (*f*) or reverse (*r*) strand. **b** Variant calling overview and workflow

**Fig. 2:**
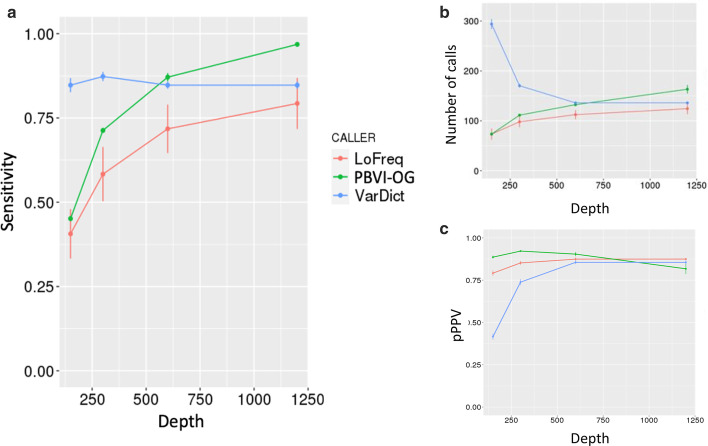
Sensitivity comparison at different depths. **a** The average sensitivity and standard error is calculated and plotted for different depths tested. **b** The average total number of variant calls and standard error is plotted. **c** The proxy positive predictive value (pPPV) and standard error is plotted for different depths tested

**Fig. 3:**
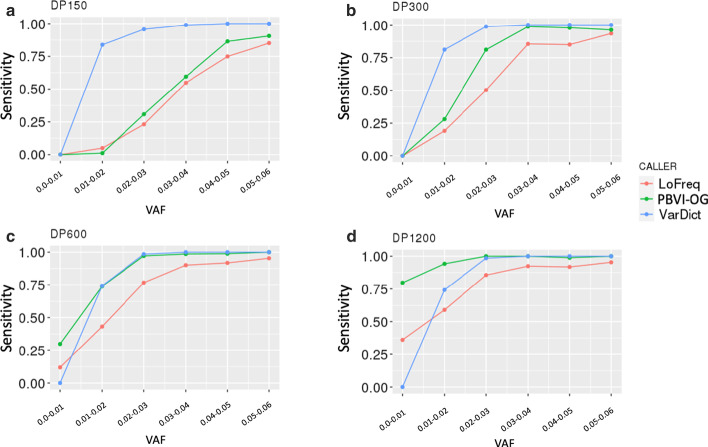
Sensitivity plot for different depths (DP) and Variant Allele Fractions (VAFs). **a**–**d** Different depths tested. DP150, DP300, DP600, DP1200 indicates sensitivity plots for 150×, 300×, 600×, and 1200× depths, respectively. The VAF range is indicated as lower bound (inclusive)- upper bound (exclusive)

The overview of the PBVI method is described in Fig. 1. We evaluated the robustness of PBVI in combination with the OverGrowth Position-Based model (PBVI-OG) for classifying low-level variants by simulating variants in three independently generated BAM files and measuring sensitivity. We introduced synthetic variants directly into our sequencing files by using SomatoSim, a SNV simulation tool (see Methods). The advantages of this approach compared to generating synthetic reads from a reference file or admixture of sequencing files are that this approach allows for error profiles to be preserved and does not limit the VAFs, locations, and number of variants that can be simulated [7]. For each BAM file, variants were simulated with a VAF between 0 and 0.06 (see Additional file 1: Table S1). Sensitivity (SEN) was used to assess variant calling throughout the paper. Additionally, we examined the proxy positive predictive value (pPPV) as an estimate to PPV. At low level VAFs it is difficult to determine truth from error in experimental data, making positive predictive value impossible to accurately assess. However, we hypothesized that as the total number of identified variants increased beyond that of the simulated variant set, this indicated a decrease in positive predictive value, thus we used the ratio of simulated variants (true positive) over the total number of variant calls as a proxy measurement for the positive predictive value. We determined the sensitivity and pPPV of PBVI-OG for identifying simulated variants and compared the results to the two unmatched mosaic variant callers, LoFreq and VarDict (see Table 1, Additional file 1: Table S2). The sensitivity of all callers is both read depth- and VAF-dependent (Figs. 2, 3, Table 1). Overall, the average sensitivity and pPPV across the simulated variant sets across all depths was PBVI-OG, SEN = 0.74, pPPV = 0.88; LoFreq, SEN = 0.62, pPPV = 0.85; VarDict, SEN = 0.85, pPPV = 0.72. While VarDict had the higest average sensitivity, it had low pPPV compared to both PBVI-OG and LoFreq due to the high number of variant calls at depths < 600 × (Fig. 2). While LoFreq had comparable pPPV to PBVI-OG, the overall sensitivity was lower than PBVI-OG. PBVI-OG had the highest average pPPV with the highest average sensitivity across the simulated variant sets.

**Table 1:** Sensitivity and proxy positive predictive value (pPPV) summarized for each caller at varying depths. The pPPV values are shown in parenthesis

	Simulation 1	Simulation 2	Simulation 3	Mean	SE
*PBVI-OG*					
150×	0.46 (0.90)	0.44 (0.88)	0.45 (0.88)	0.45 (0.89)	0.01 (0.01)
300×	0.71 (0.92)	0.72 (0.92)	0.70 (0.93)	0.71 (0.92)	0.01 (0.00)
600×	0.87 (0.91)	0.85 (0.88)	0.89 (0.92)	0.87 (0.90)_	0.01 (0.01)
1200×	0.96 (0.88)	0.96 (0.80)	0.98 (0.78)	0.97 (0.82)	0.01 (0.03)
*LoFreq*					
150×	0.55 (0.82)	0.36 (0.76)	0.31 (0.79)	0.41 (0.79)	0.07 (0.02)
300×	0.74 (0.87)	0.55 (0.83)	0.46 (0.85)	0.58 (0.85)	0.08 (0.01)
600×	0.84 (0.89)	0.71 (0.86)	0.60 (0.88)	0.71 (0.87)	0.07 (0.01)
1200×	0.93 (0.87)	0.77 (0.87)	0.67 (0.89)	0.79 (0.87)	0.08 (0.01)
*VarDict*					
150×	0.89 (0.45)	0.82 (0.40)	0.84 (0.39)	0.84 (0.42)	0.02 (0.02)
300×	0.90 (0.77)	0.86 (0.71)	0.86 (0.73)	0.87 (0.74)	0.01 (0.02)
600×	0.87 (0.87)	0.85 (0.83)	0.83 (0.87)	0.85 (0.86)	0.01 (0.01)
1200×	0.87 (0.87)	0.85 (0.83)	0.83 (0.87)	0.85 (0.86)	0.01 (0.01)

Specifically, PBVI-OG demonstrated a higher sensitivity as compared to LoFreq at all combinations of VAF and read depths except for read depth 150 ×, VAF < 0.02 (PBVI-OG, SEN = 0.01; LoFreq, SEN = 0.03). LoFreq also had higher standard error for calculated average sensitivity values at all read depths (Fig. 2a).

At VAF ≤ 0.01 and 600 × depth, PBVI-OG showed increased sensitivity compared to VarDict (PBVI-OG, SEN = 0.3; VarDict, SEN = 0), a trend that continued at 1200 × depth (PBVI-OG, SEN = 0.8; VarDict, SEN = 0) (Fig. 3c, d). VarDict showed higher sensitivity for all VAFs at depths < 600 ×, likely attributed to the high number of variant calls, showing the lowest pPPV at depths < 600 × (Fig. 2c). VarDict called more than twice the number of variants at 150 × depth as compared to 600 × depth (average number of calls of 294 vs. 136) suggesting that a high percentage of variants called by VarDict at low read depths are likely to be false positives.

### Control dataset size is important for optimizing model performance

**Fig. 4:**
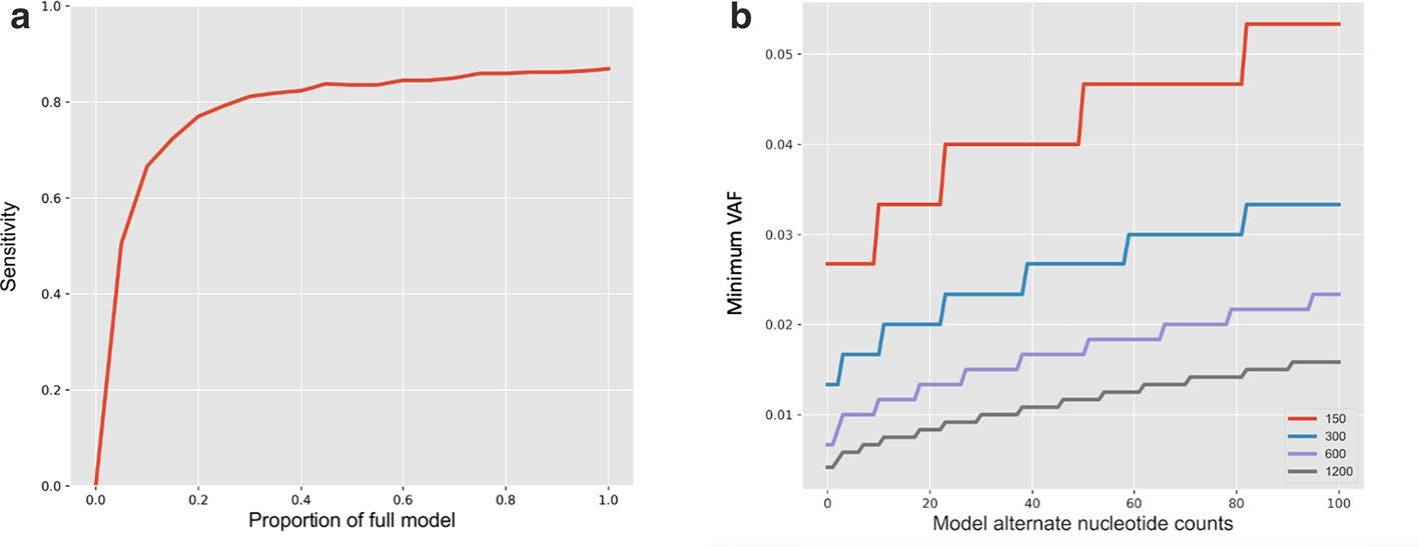
Simulation of model variables. **a** Classification performance on simulated dataset of low-level variants as function of model size. **b** Minimum variant allele fraction (VAF) as function of alternate nucleotides present in model. The line color represents depth in the test sample

To understand the effect of model size on the performance of PBVI-OG, we simulated different total nucleotide counts by scaling the OG model, thus keeping the proportion of reference to alternate nucleotides constant, and measured the effects on sensitivity of variant calling on the simulated BAM datasets (Fig. 4a). We observed a notable increase in sensitivity as a larger percentage of the full model was employed. We observed that the simulation reached comparable performance to the full model (average coverage per position 26,769) at model nucleotide counts equivalent to half of the full model. Specifically, the sensitivity calculated for this simulation when using the full model size was 0.87 and the sensitivity when using half of the full model size was 0.84.

### Alternate nucleotides in the control dataset modulate limits of detection

Next, we investigated how the presence of alternate nucleotides in the model, which would include both sequencing error and true variants in the control dataset, affected the theoretical ability of PBVI to identify variants. We determined the minimum VAF at which PBVI would call variants in relation to model alternate nucleotide counts at simulated depths specified to 150 ×, 300 ×, 600 × and 1200 ×. As expected, we observed a positive correlation (R^2^ > 0.90) of the limits of detection to the number of model alternate nucleotide counts (Fig. 4b). For a given sample read depth and model reference nucleotide count (assumed to be 26,082), the minimum VAF detectable by PBVI decreased as the number of model alternate nucleotide counts decreased. When the depth in the sample was ~ 600 × and the alternate nucleotide count in the model was set to the inherent sequencing error rate on Illumina platforms (26 out of 26,082 ≅ 0.001), the minimum VAF called by the PBVI was ~ 0.01. When we increased the alternate nucleotide counts in the model to 60 (out of 26,082), the minimum VAF detected was ~ 0.015.

These simulations also allowed us to compare the effects of variations in sample read depths on detection limits. We observed an inverse correlation (R^2^ = 0.69) of the minimum VAFs detected to the sample read depth. When the model was set to the inherent sequencing error rate on the Illumina platforms as mentioned above, the minimum VAF required to be called by the model for sample read depths of 150 ×, 300 ×, 600 ×, and 1200 × was 0.04, ~ 0.023, ~ 0.013, and ~ 0.009, respectively.

### Effects of position-based and nucleotide specific modeling

**Fig. 5:**
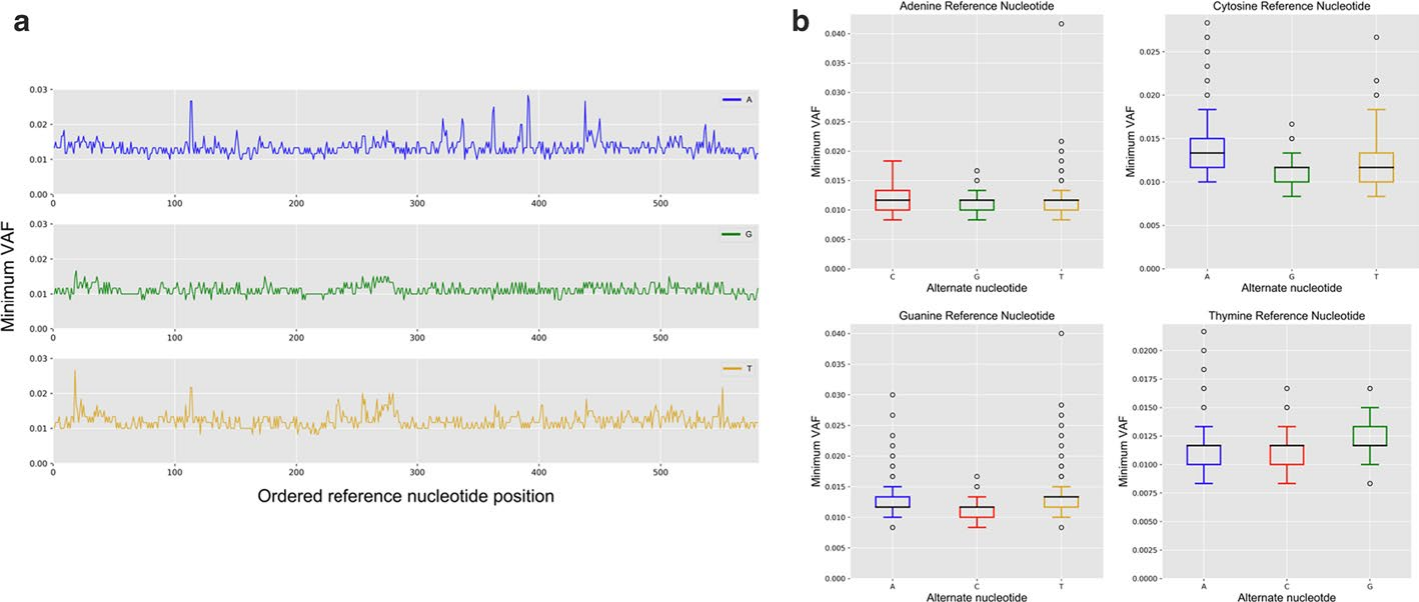
Minimum Variant Allele Fraction (VAF) for positions in *PIK3CA* with reference nucleotide cytosine required for Position-Based Variant Identification using the OverGrowth (PBVI-OG) model at 600× test sample depth. **a** Minimum VAF callable at every position where the reference nucleotide is cytosine and for each alternate nucleotide was calculated and plotted for the model. Blue (adenine), green (guanine), yellow (thymine). **b** Boxplot of minimum VAF detected for all nucleotide changes

To demonstrate the effects of position-based factors on variant calling, we determined the lower limit of variant detection for all possible variants in a single gene from our 11 gene model, *PIK3CA.* We calculated the minimum VAF detected by the model at a test depth of 600 × for each possible alternate nucleotide across the entire ORF of the gene. The results for positions with cytosine as the reference nucleotide are presented in Fig. 5a. A boxplot and summary of the mean minimum VAF detected across *PIK3CA* for specific reference nucleotide/alternate nucleotide combinations are shown in Fig. 5b and in Additional file 1: Table S3. As expected, the minimum VAF required to call a variant not only varied across positions with the same alternate nucleotide, but also across alternate nucleotides at the same position (Fig. 5a). Higher peaks observed in Fig. 5a suggested that the errors observed across the ClinSeq^®^ population were non-random, indicating position-specific errors within the cohort data. Across *PIK3CA*, we found that the minimum VAF necessary for identifying a variant was higher for C > A variants, compared to C > G or C > T and higher for T > G, compared to T > A or T > C (Fig. 5b).

### OG model performance on somatic overgrowth disorders cohort

To determine the performance of our OG model on real datasets, we performed variant calling on 27 libraries from 26 individuals with somatic overgrowth disorders using PBVI-OG, LoFreq, and VarDict. Four libraries from three individuals with known causative variants at low VAF (0.026–0.065) were run as positive controls, an additional 23 libraries were from individuals not previously studied. All results were filtered for variants with < 10 variant alleles in gnomAD v2.1.1 and variants were assessed for pathogenicity based on information in the Catalogue of Somatic Mutations in Cancer (COSMIC), ClinVar, and the literature. PBVI-OG called a total of 171 single nucleotide variants (SNVs) with a combined p-value that met our < 0.05 cut off after Bonferroni correction (1–13 SNVs per sample, average 2.3 × 10^−1^ SNV/kb). LoFreq called a total of 22 SNVs (0–5 SNVs per sample, average 2.9 × 10^−2^ SNV/kb), and VarDict called a total of 907 SNVs (1–106 SNVs per sample, average 1.2 SNV/kb). A manual review for known pathogenic variants in *PIK3CA* showed an additional variant that was not detected by any of the three callers. Variants were assessed for pathogenicity and 19 libraries from 18 individuals were determined to harbor pathogenic (P) or likely pathogenic (LP) [18] variants for somatic overgrowth disorders. PBVI-OG and VarDict called 18/19 of these variants and LoFreq called 12/19 of these variants. An additional variant with VAF below 0.01 (5/615 reads, p-value > 0.05) was identified by manual review of positions in *PIK3CA* known to contribute to somatic overgrowth. This variant was not identified by PBVI-OG as the p-value was above 0.05 after Bonferroni correction. Nine samples contained P/LP variants with VAF < 0.05, ddPCR probes were available to confirm variants in six of these samples and two variants were confirmed with a restriction enzyme digest designed to detect the known causative variant in *AKT1* for Proteus syndrome. Confirmation was not attempted for the ninth variant with VAF < 0.05 as a ddPCR probe was not readily available. A summary of library statistics, including the number of variants that were identified by each of the three callers (PBVI-OG, LoFreq and VarDict, filtered for gnomAD v2.1.1 variant allele count < 10) and average depth of coverage is presented in Additional file 1: Table S4. Variants of interest are presented in Additional file 1: Table S5.

PBVI-OG and VarDict both demonstrated high sensitivity identifying 95% (18/19) of P/LP variants, while LoFreq had a lower sensitivity identifying 63% of P/LP variants (12/19) in this cohort. A single variant with a VAF < 0.01 was identified in the data that was not called by any of the three callers. PBVI-OG had a positive predictive value of 10% with 18/177 variants being determined to be P/LP. Positive predictive value for VarDict was low at approximately 2% (18/907) while LoFreq had the highest positive predictive value of 55% (12/22). Combining sensitivity and positive predictive value the PBVI-OG model outperformed the other two callers for this dataset.

## Discussion

The identification of mosaic variants in individuals with somatic overgrowth disorders can be challenged by a low VAF that approaches the limit of sequencing error. As sequencing error is not consistent across all positions, due to the effect of position-based attributes including sequence context and mappability, we set out to improve low-level variant calling by directly modeling sequencing error from a control dataset. Our method, PBVI, compares observed nucleotide counts in pooled reads from a control dataset to nucleotide counts in a test sample to determine which alternate nucleotides are likely to be true variants. Germline variants in the control dataset can be filtered from the model if desired although we chose not to do this as our goal was to identify pathogenic variants in patients with somatic overgrowth. We reasoned that alternate nucleotides in the control dataset were either biologically real, and thus not likely to be pathogenic for overgrowth disorders, or not biologically real, and thus were a result of sequencing artifacts. PBVI does not distinguish these two types of alternate nucleotides if they are included in the control dataset and both were counted during the OG model building. While previous studies have used pooled datasets [12, 13], PBVI improved upon this by considering forward and reverse reads independently allowing for the consideration of sequence context. The underlying reliance of PBVI on p-values derived from strand-specific alternate read counts resulted in removal of variants with alternate reads limited to either the forward or reverse strands. As the forward and reverse strands have distinct sequence context preceding a position, sequencing error resulting from sequence context may be strand-specific and such positions would be filtered from the variant list.

The performance of PBVI was compared to two unmatched mosaic callers, LoFreq [17] and VarDict [16] using the OG model. PBVI performs variant identification by comparing sample data to error present in a control dataset. We therefore tested PBVI-OG using data simulated into experimental BAM files which were not sanitized for low level variants. While this method of testing performance allows for determination of sensitivity it is impossible to test for true precision as mosaic variant truth is unknown in the data. In isolation, it is impossible to determine if a variant, even one present in only one read, is a true mosaic variant or sequencing error. In place of precision one can, however, consider the number of variants that are returned in addition to the simulated variants. If we consider the simulated variants to be the variants of interest, we can ask what fraction of identified variants these represent (pPPV).

When comparing PBVI to LoFreq, PBVI-OG had higher sensitivity at almost all combinations of read depth and VAF. In our comparisons, LoFreq generally returned fewer variants overall suggesting it is a more stringent caller. At read depths of 150 ×, VarDict had higher sensitivity as compared to both PBVI-OG and LoFreq but improved sensitivity was accompanied by increased calling of non-simulated variants. As read depth increased, VarDict returned fewer non-simulated variants suggesting that the increased sensitivity of VarDict at low read depths came at the cost of specificity.

There are two model variables that can directly influence the performance of PBVI: the total nucleotide counts (model size) and alternate nucleotide counts summed across the control cohort at each position. PBVI relies on control data to model error and it is important to understand the effect of model size on performance as this dictates the amount of control data that must be available for model building. It was determined that using fifty percent of the OG model in model simulation analysis, with an average coverage of 13,385 reads per position, gave a sensitivity of 0.84 which was just slightly reduced from the sensitivity achieved using the whole model (0.87). With exome coverage of 100 × this suggests an effective model could be built from a control cohort of approximately 130 individuals. It is important to note that as this is a comparative method, variants present in the control individuals at increased VAF (either germline or common mosaic) used for model building may not be identified in the sample even if present. It is therefore imperative that the phenotypic features under investigation not reside in the control dataset. The effect of alternate nucleotides in the model was also investigated by direct modeling of variables and calculation of p-values. PBVI removes presumed error by comparing alternate nucleotide counts in sample and control data. As alternate nucleotide counts increase in the model, either due to recurring error in the control dataset or due to germline occurrence of the variant, the VAF required for a variant to be called in the sample data will increase.

The PBVI method is based on the assumption that each position in the genome has a position-based and alternate nucleotide-specific error associated with it, with some positions having low error and others having high error as compared to the average error for Illumina sequencing of ~ 0.1%. Error is expected to include misincorporation, based on the underlying nucleotide with known rates of preferential base misincorporations and sequence context. Systematic error is also expected to include mapping error. In order to understand the effect of underlying error on the ability of PBVI-OG to call variants we assessed the VAF required at each position in *PIK3CA* for variant identification at 600 × read depth (Fig. 5). The limit of mosaic variant calling is higher for C > A variants which is consistent with previously known preferential C > A nucleotide misincorporation. We found that the observed trends in the mean minimum VAF across positions in *PIK3CA* mirrored the preferentially misincorporated nucleotides reported in the literature (A > C, C > A, G > T), but not T > G (Fig. 5b). Note that heterozygous variants, including common SNPs, were not removed from the OG model. Including such variants in the model limits the calling of low-level mosaic variants at these positions as higher alternate reads in the model correlate with higher detectable VAFs, as shown in Fig. 4.

As PBVI-OG was designed for low-level mosaic variant detection in individuals with overgrowth disorders, it was piloted on a set of 27 samples from 26 individuals. PBVI-OG and VarDict identified 18/19 variants, with a final variant identified through manual review for known pathogenic variants in *PIK3CA*. LoFreq identified 12/19 variants. When clinically analyzing variants both sensitivity and specificity are important as identified variants need to be assessed for pathogenicity. When a caller has reduced specificity, the result is extra time and effort for variant confirmation and/or pathogenicity assessment. PBVI-OG returned 171 variants that passed filter compared to 22 variants for LoFreq and 907 variants for VarDict. The cost of LoFreq only returning 22 variants was reduced sensitivity which is not a desirable trade off. VarDict, however, returned over five times the number of variants as compared to PBVI which increases the downstream analysis of variant pathogenicity.

## Conclusions

In this work, we developed an approach for low-level variant calling in non-matched samples that can be adapted for a wide range of mosaic diseases. PBVI-OG had the highest average sensitivity and pPPV across the simulated variant sets, and was shown to have better sensitivity at low VAF and high read depth as compared to LoFreq and VarDict. As well, PBVI-OG demonstrated a better combination of both sensitivity and proxy positive predictive value as compared to LoFreq and VarDict when used for analysis of 27 overgrowth samples. The work presented here is based on the Illumina sequencing platform and the generalizability of this method across platforms and chemistries is not known. As the control data were generated on an Illumina HiSeq instrument and sample data was generated on an Illumina MiSeq instrument, our data suggest that trends in error across these two platforms are consistent. It is possible that using control data generated on the same instrument would have resulted in improved performance. Lastly, our measures of caller performance were somewhat limited by a lack of large orthogonally validated, low-level variant datasets. Nevertheless, using a position-based, nucleotide-specific modeling approach, we demonstrated that we can effectively detect low-level variants with improvement in sensitivity and positive predictive value as compared to other unmatched variant callers.

## Methods

### Control dataset

Exome data generated from the ClinSeq^®^ A2 cohort was used as the control dataset for this study [19]. All 502 individuals in this cohort were free from apparent somatic overgrowth disorders at the time of enrollment. Genomic DNA was isolated from peripheral blood using standard protocols (Gentra Blood Kit; Qiagen, Gaithersburg, MD). Sequencing was performed at the NIH Intramural Sequencing Center. Briefly, sequencing libraries were generated from 100 ng of genomic DNA using the Accel-NGS 2S DNA Library Kit (Swift Biosciences, Ann Arbor, MI) on a Biomek FX robot (Beckman Coulter, Eldersburg, MD) with median insert size of 250 bp. Libraries were then dual-indexed, pooled in groups of eight, and enriched with the xGen Exome Research Panel (IDT, Rockville, MD). Sequencing was performed on the HiSeq 4000 (Illumina, San Diego, CA). After demultiplexing, reads were processed according to the GATK best practices workflow using GATK v4.0.2.0 [20]. Reads were filtered for mapping quality > 20. Average sequencing coverage was 64 ×.

## Somatic overgrowth dataset

Next generation sequencing data from 26 individuals with apparent somatic overgrowth disorders were evaluated for this study, which included individuals diagnosed with Proteus syndrome (MIM:176920) [21] and other segmental overgrowth phenotypes [22]. Three individuals had known variants at the time of the study and the remaining 23 individuals were untested for mosaic variants. Affected areas of the body were biopsied and DNA was extracted using the Gentra Puregene kit (Qiagen, Germantown, MD) according to manufacturer’s directions. In one case, two distinct samples were processed for a single individual. Dual-indexed sequencing libraries were prepared according to manufacturer’s protocols using the NEBNext dsDNA Fragmentase, NEBNext Ultra II DNA Library Prep Kit for Illumina, and NEBNext Multiplex Oligos for Illumina kits (New England Biolabs, Beverly, MA). An 11 gene custom capture panel (OverGrowth v1) was designed to include genes known to be causative or potentially causative for somatic overgrowth disorders (Integrated DNA Technologies (IDT), Coralville, IA) and included the following genes: *AKT1* (MIM:164730)*, FGFR1* (MIM:136350)*, GNAQ* (MIM:1600998)*, KRAS* (MIM:190070)*, PDGFRB* (MIM:173410)*, PHLPP1* (MIM:1609396)*, PIK3CA* (MIM:171834)*, PIK3R1* (MIM:171833)*, PIK3R2* (MIM:603157)*, RASA1* (MIM:139150)*, TEK* (MIM:600221)*.* Briefly, ~ 1 µg of genomic DNA was fragmented, purified with the MinElute PCR Purification Kit (Qiagen), end-prepped and adaptor-ligated, size-selected for 320 bp fragments with AMPure XP beads (Beckman Coulter, Sykesville, MD), and dual-indexed during seven cycles of PCR enrichment. After library preparation, libraries were quantified with Qubit fluorometry (Thermofisher, Rockville, MD) and pooled with 100 ng of each sample in sets of eight to ten samples. Capture of DNA fragments was performed using the xGen Lockdown Probe Pool and xGen lockdown Reagents according to manufacturer’s protocols (IDT). All libraries were run on an Illumina MiSeq v2 nano flow cell at 10 ppM loading concentration. In total, 27 libraries were sequenced. Additionally, three samples used for simulated dataset creation underwent a second round of library preparation with an updated capture panel (OverGrowth v2) designed to include 16 genes (additional genes: *AKT2* (MIM:164731)*, AKT3* (MIM:611223), *GNAS* (MIM:139320), *MTOR* (MIM:601231), *PTEN* (MIM:601728)), and libraries were sequenced on a v2 standard flow cell to generate high depth sequence data.

After sequencing, demultiplexing was performed on-machine by Illumina’s MiSeq Reporter v2.6 software. Fastq files for each sample were then processed according to GATK’s best practices workflow using GATK v3.8–1.0 with Base Recalibration step removed as recommended for smaller dataset (< 100 M bases) [20]. Reads were filtered for mapping quality > 20.

The ClinSeq^®^ and overgrowth studies were approved by Institutional Review Boards at the National Institutes of Health.

### Overgrowth model generation

PBVI compares alternate nucleotide counts in control sequence data to alternate nucleotide counts in test data to differentiate likely error from likely true variation. PBVI requires alternate nucleotides at each position in the control data to be arrayed in a table format, this table is referred to as the “model”. For these analyses the model was restricted to positions targeted by the OverGrowth v1 panel. A RefSeq BED file was downloaded from the UCSC table browser containing genomic coordinates for coding exons of the 11 genes in the custom capture OverGrowth v1 panel. The BED file was a total of 29,083 bp. Nucleotide counts were extracted from each BAM file in the control dataset at positions that overlapped the BED file using a custom python script. The custom python script produced a text file wherein each row is a position in the BED file and the columns contain the sum of nucleotide counts across all BAM files. This python script is parametrized by the minimum base quality of nucleotides to count *b*, the minimum mapping quality of reads to be considered for counting *m*, and the number of samples to use during model building *s*. Forward and reverse nucleotides are counted separately and then summed to also obtain the total nucleotide counts at each position. The script was parametrized with *b* = 0 and *s* = 502. The total number of positions included in the model was 28,119. For each position, observed counts for each nucleotide (A, G, C, and T) were summed across the control dataset. To incorporate strand-awareness in our approach, the position information was stored separately in matrices to account for the forward or reverse strand of a read. We refer to the model as OverGrowth Position-Based (OG) model (Fig. 1). PBVI python scripts were run using python 2.7. All relevant files and custom scripts are available at github.com/BieseckerLab/PBVI.

### Position-based variant identification (PBVI)

Variant calling was based on a 2 × 2 Fisher’s exact test in which the alternate and reference nucleotide counts of candidate variants in samples were compared to alternate and reference nucleotide counts in the model. An input file was defined as a sample BAM file. For each input file, nucleotide counts were generated at every position that overlapped 28,119 positions in the OG model. The candidate variant was defined by the alternate nucleotide with the largest VAF. Candidate variant nucleotide counts were then matched to model nucleotide counts based on chromosome, position, nucleotide identity, and by strand. For each candidate variant, two separate Fisher’s exact tests were performed on nucleotide counts found on each strand and combined into a single p-value using Stouffer’s method [23]. The Bonferroni multiple testing correction was applied with the number of tests set to the total number of positions in the model (28,119). Variants with Bonferroni corrected p-value of < 0.05 were classified as called variants.

As the performance of PBVI is dependent on the model it is appropriate to detail the model that is used for any analysis. In this manuscript, except where specified, PBVI was used in conjunction with the OG model and is notated as PBVI-OG.

## Comparison analysis

For all comparisons, LoFreq and VarDict were run as recommended with default parameters.

### Simulated datasets

Simulated BAM files were generated using the somatic SNV simulation tool, SomatoSim (http://www.github.com/BieseckerLab/SomatoSim). In brief, SomatoSim introduces variants into an existing, experimentally generated, BAM file at positions specified in an input BED file [32]. Our experimentally-generated BAM files were from three overgrowth samples run on a standard v2 flow cell. We compiled a variant list that consisted of confirmed somatic missense variants from COSMIC v89 [24]. The coordinates of COSMIC variants were converted to hg19 using PyLiftover, annotated using Annovar [25]. The variants then were filtered to exclude variants found in gnomAD v2.1.1 [26], and overlapping our 11 gene custom capture panel.

For each of three different experimentally generated BAM files (average read depth 969 ×), we targeted to simulate ~ 0.5% of the total available locations. Approximately 150 variants were randomly selected from our variant list and used to generate a unique input BED file for SomatoSim. The resulting VAFs of the simulated variants ranged from 0.0 to 0.06 and were binned into 0.01 ranges.

To assess the impact of sample read depth on sensitivity, additional BAM files at varying depths were derived from the three simulated BAM files to compare the effect of depths on variant calling. For each experimentally-generated BAM file, we generated four different simulated BAM files, where the average coverage across the simulated variant positions was 150 ×, 300 ×, 600 ×, and 1200 ×. The 150 ×, 300 ×, and 600 × BAM files were generated using the down-sampling feature of SomatoSim (option *–down-sample*) and the 1200 × BAM file was generated by merging the 600 × BAM file with itself using SAMtools [27]. The SomatoSim option *–random-seed* was set to 0. The simulated variants in the 12 total BAM files are summarized in Additional file 1: Table S1.

### Effects of the OG model size on classification performance

The effect of the model size was investigated using process described above. The OG model was scaled from 0 to 1.0 times its original size, with an increment size of 0.05. For example, if the original alternate and reference model nucleotide counts at a given position were 10 and 1000, then a scaling factor of 0.1 would modify these values to 1 and 100, respectively. Variants in the three simulated BAM files were recalled using the scaled OG models and sensitivity was recalculated.

### Effect of model alternate nucleotide counts

We next evaluated the interplay of model VAF, sample read depth, and minimum detectable sample VAF with optimal conditions. For these analyses the OG model reference count was set to 26,082 (the mean model reference nucleotide counts in the 11 genes), the OG model alternate nucleotide count was varied from 0 to 100, and the sample test depth was set to 150, 300, 600 or 1200. Nucleotide counts were distributed evenly between forward and reverse strands to simulate variants supported by both strands and to control for the effect of strand bias on variant calling for the purpose of studying the relationships between variables mentioned above. In order to determine the minimum sample VAF that resulted in a significant call for each combination of model alternate nucleotide count and sample read depth a 2 × 2 contingency table was constructed to test our test cases (test alternate nucleotide count (*a*), test reference nucleotide count (*b*)) against the OG model (OG model alternate nucleotide count (*c*), OG model reference nucleotide count (*d*)). The following were the values for each variable: *a* was initialized at one with an increment step of one until significant p-value was reached; *b* = (*test depth* − *a*) with test depth = 150, 300, 600, 1200; *c* was from 1 to 100; and *d* = 28,119, which was the mean reference nucleotide count for all the positions in the OG model. For each value *c*, we performed a 2 × 2 Fisher’s exact test on the contingency table and the value for the test alternate nucleotide count (*a*) was iteratively increased by one until the resulting p-value was significant after Bonferroni multiple testing correction. All nucleotide counts in the contingency table were halved such that the resulting forward and reverse nucleotide counts tested represented an even distribution between strands. The VAF associated with the significant test alternate nucleotide count was then calculated and recorded. This process continued for every combination of test depth and OG model alternate nucleotide count tested.

### OG model-based variant detection limits in PIK3CA

To understand position-specific, nucleotide-dependent errors in the model, we followed a process similar to that above to calculate the model-based minimum VAF detected for every combination of reference nucleotide and alternate nucleotide at all positions in *PIK3CA* at a test sample depth of 600 ×. For every alternate nucleotide at every position in *PIK3CA* that is in our OG model, we performed two separate 2 × 2 Fisher’s exact tests, one for the forward strand and one for the reverse strand, then combined the p-values (using Stouffer’s method) when determining significance. When the p-value first becomes significant, the VAF was computed as the quotient of the test alternate nucleotide count and the depth, which was fixed at 600. To elaborate, in the forward strand 2 × 2 contingency table, *a* = an integer representing the test alternate nucleotide count that is initialized at one, *b* = *(300* − *a)*, *c* = the OG model alternate nucleotide count for the given position, and *d* = the OG model reference nucleotide count for the given position. For the reverse strand 2 × 2 contingency table, *a* is initialized at zero. Additionally, we alternate iteratively increasing the test alternate nucleotide count, *a*, between the forward and reverse strand, starting with the reverse strand. To examine locations with alternate nucleotides in the model arising from sequencing errors and not due to heterozygous calls, 96 locations with known heterozygous variants in the cohort were removed from the analysis. We then plotted the minimum VAF that we computed at each location in *PIK3CA* for all combinations of reference and alternate nucleotides.

### Evaluating variant calling algorithms

To assess variant calling methods, we used sensitivity to measure detection of simulated variants throughout the study. Sensitivity was calculated, where

$$sensitivity=\frac{true\;positives}{total\;simulated\;variants}$$

True positives were defined as simulated variants that were identified by a variant calling algorithm. The average sensitivity, standard error, and confidence interval was calculated for the three simulated BAM files at each depth using R library Rmisc. While we used sensitivity as the main metric to assess and compare performance, we used proxy positive predictive value (pPPV), defined by true positives over all variant calls, to estimate the PPV. We term this estimation as proxy PPV as we could not rule out the possibility of identified variants beyond the simulated variants being true mosaic variants in the experimental BAM files used for creation of the simulated files.

All plots in this study were generated using R package ggplot2 [28] and Python matplotlib [29].

## Variant calling in 26 Proteus/Somatic Overgrowth patients

BAM files were generated on 27 samples from 26 individuals with somatic overgrowth disorders and variants were called with PBVI-OG, LoFreq, and VarDict [16, 17]. Additionally, positions for known pathogenic variants in *PIK3CA* were assessed manually. Four samples from three individuals were known positive controls, the remaining 23 samples had not been previously tested. All called variants were assessed for pathogenicity. Nonsynonymous variants were first filtered for rarity in gnomAD v2.1.1 (< 10 alleles) and then assessed for pathogenicity based on data found in COSMIC and ClinVar [30]. Three individuals were included as positive controls with previously identified mosaic variants.

### Experimental validation

The known pathogenic *AKT1* c.49G > A p.(Glu17Lys) variant was confirmed using a described assay [21]. Briefly, the region surrounding NM_001014431.1(*AKT1*): c.49G > A was PCR-amplified using fluorescently-labeled engineered primers that create an *MboII* site in the presence of the variant. The amplified fragments were digested with *MboII* and products were detected on an ABI 3130xl. All other variants assessed to be pathogenic or likely pathogenic with a VAF > 0.15 were confirmed using Sanger sequence analyses. This threshold was based on prior work suggesting that Sanger sequence analysis can detect variant alleles with a VAF > 0.15 (unpubished). Known pathogenic *PIK3CA* variants (c.3140A > G; p.(His1047Arg), c.1633G > A; p.(Glu545Lys), and c.1624G > A; p.(Glu542Lys), reference NM_006218.3) with VAF < 0.05 were confirmed using digital droplet polymerase chain reaction (ddPCR) as described [31]. Probes were purchased from Biorad, Hercules, CA and samples were run in triplicate. A sample was considered confirmed if > 3 droplets were variant-positive. For all confirmations both positive and negative controls were run.

## Supplementary Information

**Additional file 1.** **Table S1**. Summary of simulated variants. VAFs were binned into 0.01, expressed as lower limit (inclusive)-upper limit (exclusive). **Table S2**. Sensitivity calculated for VAFs tested. VAFs were binned into 0.01, expressed as lower limit (inclusive)-upper limit (exclusive). **Table S3**. Mean minimum VAF detected for every combination of reference allele and alternate nucleotide in *PIK3CA* at 600 × test sample depth. **Table S4**. Number of variants identified by PBVI-OG, LoFreq, and Vardict variant callers. **Table S5**. Variants of interest identified in 27 overgrowth samples.

## Data Availability

The datasets generated and/or analyzed during the current study are available in github repository at (github.com/BieseckerLab/PBVI), in dbGaP repository, ClinSeq^®^ A2 cohort (phs000971, https://www.ncbi.nlm.nih.gov/projects/gap/cgi-bin/study.cgi?study_id=phs000971.v2.p1) and overgrowth cohort (phs002006, https://www.ncbi.nlm.nih.gov/projects/gap/cgi-bin/study.cgi?study_id=phs002006.v2.p1). Data for samples PS105.3_TL and PS105.3_TR were not submitted to dbGaP as these were historical samples without consent for deposition of sequence data.

## Abbreviations

NGS: Next-generation sequencing
SNV: Single nucleotide variant
PBVI: Position based variant identification
OG: Overgrowth
VAF: Variant allele fraction
SNP: Single nucleotide polymorphisms
COSMIC: Catalogue of somatic mutations in cancer
ddPCR: Digital droplet polymerase chain reaction
SEN: Sensitivity
