# Supplementary material for: Low-level variant calling for non-matched samples using a position-based and nucleotide-specific approach
Source: BMC Bioinformatics. 2021 Apr 8;22:181. doi: 10.1186/s12859-021-04090-y (PMC8028235; doi:10.1186/s12859-021-04090-y)
Supplement: Supplementary file 1 — Additional file 1. Table S1. Summary of simulated variants. VAFs were binned into 0.01, expressed as lower limit (inclusive)-upper limit (exclusive). Table S2. Sensitivity calculated for VAFs tested. VAFs were binned into 0.01, expressed as lower limit (inclusive)-upper limit (exclusive). Table S3. Mean minimum VAF detected for every combination of reference allele and alternate nucleotide in PIK3CA at 600 × test sample depth. Table S4. Number of variants identified by PBVI-OG, LoFreq, and Vardict variant callers. Table S5. Variants of interest identified in 27 overgrowth samples. [file 12859_2021_4090_MOESM1_ESM.docx]

**Supplementary data**

All custom scripts used in this work can be found at github.com/BieseckerLab/.

Supplementary Table 1. Summary of simulated variants. Variant allele fractions (VAFs) were binned into 0.01, expressed as lower limit (inclusive)-upper limit (exclusive).

| Depth | VAF | Simulation 1 | Simulation 2 | Simulation 3 | Total |
| --- | --- | --- | --- | --- | --- |
| 150X | 0.00-0.01 | 12 | 19 | 18 | 49 |
|  | 0.01-0.02 | 27 | 26 | 28 | 81 |
|  | 0.02-0.03 | 33 | 27 | 22 | 82 |
|  | 0.03-0.04 | 21 | 32 | 34 | 87 |
|  | 0.04-0.05 | 34 | 25 | 32 | 91 |
|  | 0.05-0.06 | 15 | 14 | 13 | 42 |
|  | **Total** | **142** | **143** | **147** | **432** |
| 300X | 0.00-0.01 | 9 | 15 | 13 | 37 |
|  | 0.01-0.02 | 30 | 28 | 32 | 90 |
|  | 0.02-0.03 | 32 | 29 | 28 | 89 |
|  | 0.03-0.04 | 28 | 33 | 29 | 90 |
|  | 0.04-0.05 | 25 | 22 | 32 | 79 |
|  | 0.05-0.06 | 16 | 18 | 13 | 47 |
|  | **Total** | **140** | **145** | **147** | **432** |
| 600X | 0.00-0.01 | 14 | 13 | 13 | 40 |
|  | 0.01-0.02 | 23 | 29 | 31 | 83 |
|  | 0.02-0.03 | 32 | 25 | 22 | 79 |
|  | 0.03-0.04 | 24 | 24 | 29 | 77 |
|  | 0.04-0.05 | 24 | 29 | 34 | 87 |
|  | 0.05-0.06 | 18 | 16 | 12 | 46 |
|  | **Total** | **135** | **136** | **141** | **412** |
| 1,200X | 0.00-0.01 | 14 | 13 | 13 | 40 |
|  | 0.01-0.02 | 23 | 29 | 31 | 83 |
|  | 0.02-0.03 | 32 | 25 | 22 | 79 |
|  | 0.03-0.04 | 24 | 24 | 29 | 77 |
|  | 0.04-0.05 | 24 | 29 | 34 | 87 |
|  | 0.05-0.06 | 18 | 16 | 12 | 46 |
|  | **Total** | **135** | **136** | **141** | **412** |

Supplementary Table 2. Sensitivity calculated for variant allele fractions (VAFs) tested. VAFs were binned into 0.01, expressed as lower limit (exclusive)-upper limit (inclusive).

| Method | Simulation Sample | VAF | # Called Variants | # Simulated Variants | Sensitivity | Depth |
| --- | --- | --- | --- | --- | --- | --- |
| LoFreq | 1 | 0.0-0.01 | 0 | 12 | 0.00 | 150X |
| LoFreq | 1 | 0.01-0.02 | 2 | 27 | 0.07 | 150X |
| LoFreq | 1 | 0.02-0.03 | 15 | 33 | 0.45 | 150X |
| LoFreq | 1 | 0.03-0.04 | 16 | 21 | 0.76 | 150X |
| LoFreq | 1 | 0.04-0.05 | 31 | 34 | 0.91 | 150X |
| LoFreq | 1 | 0.05-0.06 | 14 | 15 | 0.93 | 150X |
| LoFreq | 1 | 0.0-0.01 | 0 | 9 | 0.00 | 300X |
| LoFreq | 1 | 0.01-0.02 | 12 | 30 | 0.40 | 300X |
| LoFreq | 1 | 0.02-0.03 | 27 | 32 | 0.84 | 300X |
| LoFreq | 1 | 0.03-0.04 | 26 | 28 | 0.93 | 300X |
| LoFreq | 1 | 0.04-0.05 | 23 | 25 | 0.92 | 300X |
| LoFreq | 1 | 0.05-0.06 | 15 | 16 | 0.94 | 300X |
| LoFreq | 1 | 0.0-0.01 | 5 | 14 | 0.36 | 600X |
| LoFreq | 1 | 0.01-0.02 | 15 | 23 | 0.65 | 600X |
| LoFreq | 1 | 0.02-0.03 | 32 | 32 | 1.00 | 600X |
| LoFreq | 1 | 0.03-0.04 | 23 | 24 | 0.96 | 600X |
| LoFreq | 1 | 0.04-0.05 | 22 | 24 | 0.92 | 600X |
| LoFreq | 1 | 0.05-0.06 | 17 | 18 | 0.94 | 600X |
| LoFreq | 1 | 0.0-0.01 | 13 | 14 | 0.93 | 1,200X |
| LoFreq | 1 | 0.01-0.02 | 19 | 23 | 0.83 | 1,200X |
| LoFreq | 1 | 0.02-0.03 | 32 | 32 | 1.00 | 1,200X |
| LoFreq | 1 | 0.03-0.04 | 23 | 24 | 0.96 | 1,200X |
| LoFreq | 1 | 0.04-0.05 | 22 | 24 | 0.92 | 1,200X |
| LoFreq | 1 | 0.05-0.06 | 17 | 18 | 0.94 | 1,200X |
| LoFreq | 2 | 0.0-0.01 | 0 | 19 | 0.00 | 150X |
| LoFreq | 2 | 0.01-0.02 | 2 | 26 | 0.08 | 150X |
| LoFreq | 2 | 0.02-0.03 | 4 | 27 | 0.15 | 150X |
| LoFreq | 2 | 0.03-0.04 | 16 | 32 | 0.50 | 150X |
| LoFreq | 2 | 0.04-0.05 | 17 | 25 | 0.68 | 150X |
| LoFreq | 2 | 0.05-0.06 | 13 | 14 | 0.93 | 150X |
| LoFreq | 2 | 0.0-0.01 | 0 | 15 | 0.00 | 300X |
| LoFreq | 2 | 0.01-0.02 | 4 | 28 | 0.14 | 300X |
| LoFreq | 2 | 0.02-0.03 | 12 | 29 | 0.41 | 300X |
| LoFreq | 2 | 0.03-0.04 | 29 | 33 | 0.88 | 300X |
| LoFreq | 2 | 0.04-0.05 | 18 | 22 | 0.82 | 300X |
| LoFreq | 2 | 0.05-0.06 | 17 | 18 | 0.94 | 300X |
| LoFreq | 2 | 0.0-0.01 | 0 | 13 | 0.00 | 600X |
| LoFreq | 2 | 0.01-0.02 | 13 | 29 | 0.45 | 600X |
| LoFreq | 2 | 0.02-0.03 | 20 | 25 | 0.80 | 600X |
| LoFreq | 2 | 0.03-0.04 | 22 | 24 | 0.92 | 600X |
| LoFreq | 2 | 0.04-0.05 | 26 | 29 | 0.90 | 600X |
| LoFreq | 2 | 0.05-0.06 | 16 | 16 | 1.00 | 600X |
| LoFreq | 2 | 0.0-0.01 | 2 | 13 | 0.15 | 1,200X |
| LoFreq | 2 | 0.01-0.02 | 18 | 29 | 0.62 | 1,200X |
| LoFreq | 2 | 0.02-0.03 | 21 | 25 | 0.84 | 1,200X |
| LoFreq | 2 | 0.03-0.04 | 22 | 24 | 0.92 | 1,200X |
| LoFreq | 2 | 0.04-0.05 | 26 | 29 | 0.90 | 1,200X |
| LoFreq | 2 | 0.05-0.06 | 16 | 16 | 1.00 | 1,200X |
| LoFreq | 3 | 0.0-0.01 | 0 | 18 | 0.00 | 150X |
| LoFreq | 3 | 0.01-0.02 | 0 | 28 | 0.00 | 150X |
| LoFreq | 3 | 0.02-0.03 | 2 | 22 | 0.09 | 150X |
| LoFreq | 3 | 0.03-0.04 | 13 | 34 | 0.38 | 150X |
| LoFreq | 3 | 0.04-0.05 | 21 | 32 | 0.66 | 150X |
| LoFreq | 3 | 0.05-0.06 | 9 | 13 | 0.69 | 150X |
| LoFreq | 3 | 0.0-0.01 | 0 | 13 | 0.00 | 300X |
| LoFreq | 3 | 0.01-0.02 | 1 | 32 | 0.03 | 300X |
| LoFreq | 3 | 0.02-0.03 | 7 | 28 | 0.25 | 300X |
| LoFreq | 3 | 0.03-0.04 | 22 | 29 | 0.76 | 300X |
| LoFreq | 3 | 0.04-0.05 | 26 | 32 | 0.81 | 300X |
| LoFreq | 3 | 0.05-0.06 | 12 | 13 | 0.92 | 300X |
| LoFreq | 3 | 0.0-0.01 | 0 | 13 | 0.00 | 600X |
| LoFreq | 3 | 0.01-0.02 | 6 | 31 | 0.19 | 600X |
| LoFreq | 3 | 0.02-0.03 | 11 | 22 | 0.50 | 600X |
| LoFreq | 3 | 0.03-0.04 | 24 | 29 | 0.83 | 600X |
| LoFreq | 3 | 0.04-0.05 | 32 | 34 | 0.94 | 600X |
| LoFreq | 3 | 0.05-0.06 | 11 | 12 | 0.92 | 600X |
| LoFreq | 3 | 0.0-0.01 | 0 | 13 | 0.00 | 1,200X |
| LoFreq | 3 | 0.01-0.02 | 10 | 31 | 0.32 | 1,200X |
| LoFreq | 3 | 0.02-0.03 | 16 | 22 | 0.73 | 1,200X |
| LoFreq | 3 | 0.03-0.04 | 26 | 29 | 0.90 | 1,200X |
| LoFreq | 3 | 0.04-0.05 | 32 | 34 | 0.94 | 1,200X |
| LoFreq | 3 | 0.05-0.06 | 11 | 12 | 0.92 | 1,200X |
| PBVI-OG | 1 | 0.0-0.01 | 0 | 12 | 0.00 | 150X |
| PBVI-OG | 1 | 0.01-0.02 | 0 | 27 | 0.00 | 150X |
| PBVI-OG | 1 | 0.02-0.03 | 9 | 33 | 0.27 | 150X |
| PBVI-OG | 1 | 0.03-0.04 | 12 | 21 | 0.57 | 150X |
| PBVI-OG | 1 | 0.04-0.05 | 32 | 34 | 0.94 | 150X |
| PBVI-OG | 1 | 0.05-0.06 | 13 | 15 | 0.87 | 150X |
| PBVI-OG | 1 | 0.0-0.01 | 0 | 9 | 0.00 | 300X |
| PBVI-OG | 1 | 0.01-0.02 | 8 | 30 | 0.27 | 300X |
| PBVI-OG | 1 | 0.02-0.03 | 24 | 32 | 0.75 | 300X |
| PBVI-OG | 1 | 0.03-0.04 | 27 | 28 | 0.96 | 300X |
| PBVI-OG | 1 | 0.04-0.05 | 25 | 25 | 1.00 | 300X |
| PBVI-OG | 1 | 0.05-0.06 | 16 | 16 | 1.00 | 300X |
| PBVI-OG | 1 | 0.0-0.01 | 5 | 14 | 0.36 | 600X |
| PBVI-OG | 1 | 0.01-0.02 | 15 | 23 | 0.65 | 600X |
| PBVI-OG | 1 | 0.02-0.03 | 32 | 32 | 1.00 | 600X |
| PBVI-OG | 1 | 0.03-0.04 | 23 | 24 | 0.96 | 600X |
| PBVI-OG | 1 | 0.04-0.05 | 24 | 24 | 1.00 | 600X |
| PBVI-OG | 1 | 0.05-0.06 | 18 | 18 | 1.00 | 600X |
| PBVI-OG | 1 | 0.0-0.01 | 13 | 14 | 0.93 | 1,200X |
| PBVI-OG | 1 | 0.01-0.02 | 19 | 23 | 0.83 | 1,200X |
| PBVI-OG | 1 | 0.02-0.03 | 32 | 32 | 1.00 | 1,200X |
| PBVI-OG | 1 | 0.03-0.04 | 24 | 24 | 1.00 | 1,200X |
| PBVI-OG | 1 | 0.04-0.05 | 24 | 24 | 1.00 | 1,200X |
| PBVI-OG | 1 | 0.05-0.06 | 18 | 18 | 1.00 | 1,200X |
| PBVI-OG | 2 | 0.0-0.01 | 0 | 19 | 0.00 | 150X |
| PBVI-OG | 2 | 0.01-0.02 | 0 | 26 | 0.00 | 150X |
| PBVI-OG | 2 | 0.02-0.03 | 9 | 27 | 0.33 | 150X |
| PBVI-OG | 2 | 0.03-0.04 | 20 | 32 | 0.63 | 150X |
| PBVI-OG | 2 | 0.04-0.05 | 21 | 25 | 0.84 | 150X |
| PBVI-OG | 2 | 0.05-0.06 | 13 | 14 | 0.93 | 150X |
| PBVI-OG | 2 | 0.0-0.01 | 0 | 15 | 0.00 | 300X |
| PBVI-OG | 2 | 0.01-0.02 | 10 | 28 | 0.36 | 300X |
| PBVI-OG | 2 | 0.02-0.03 | 24 | 29 | 0.83 | 300X |
| PBVI-OG | 2 | 0.03-0.04 | 33 | 33 | 1.00 | 300X |
| PBVI-OG | 2 | 0.04-0.05 | 22 | 22 | 1.00 | 300X |
| PBVI-OG | 2 | 0.05-0.06 | 16 | 18 | 0.89 | 300X |
| PBVI-OG | 2 | 0.0-0.01 | 2 | 13 | 0.15 | 600X |
| PBVI-OG | 2 | 0.01-0.02 | 22 | 29 | 0.76 | 600X |
| PBVI-OG | 2 | 0.02-0.03 | 24 | 25 | 0.96 | 600X |
| PBVI-OG | 2 | 0.03-0.04 | 24 | 24 | 1.00 | 600X |
| PBVI-OG | 2 | 0.04-0.05 | 28 | 29 | 0.97 | 600X |
| PBVI-OG | 2 | 0.05-0.06 | 16 | 16 | 1.00 | 600X |
| PBVI-OG | 2 | 0.0-0.01 | 9 | 13 | 0.69 | 1,200X |
| PBVI-OG | 2 | 0.01-0.02 | 29 | 29 | 1.00 | 1,200X |
| PBVI-OG | 2 | 0.02-0.03 | 25 | 25 | 1.00 | 1,200X |
| PBVI-OG | 2 | 0.03-0.04 | 24 | 24 | 1.00 | 1,200X |
| PBVI-OG | 2 | 0.04-0.05 | 28 | 29 | 0.97 | 1,200X |
| PBVI-OG | 2 | 0.05-0.06 | 16 | 16 | 1.00 | 1,200X |
| PBVI-OG | 3 | 0.0-0.01 | 0 | 18 | 0.00 | 150X |
| PBVI-OG | 3 | 0.01-0.02 | 1 | 28 | 0.04 | 150X |
| PBVI-OG | 3 | 0.02-0.03 | 7 | 22 | 0.32 | 150X |
| PBVI-OG | 3 | 0.03-0.04 | 20 | 34 | 0.59 | 150X |
| PBVI-OG | 3 | 0.04-0.05 | 26 | 32 | 0.81 | 150X |
| PBVI-OG | 3 | 0.05-0.06 | 12 | 13 | 0.92 | 150X |
| PBVI-OG | 3 | 0.0-0.01 | 0 | 13 | 0.00 | 300X |
| PBVI-OG | 3 | 0.01-0.02 | 7 | 32 | 0.22 | 300X |
| PBVI-OG | 3 | 0.02-0.03 | 24 | 28 | 0.86 | 300X |
| PBVI-OG | 3 | 0.03-0.04 | 29 | 29 | 1.00 | 300X |
| PBVI-OG | 3 | 0.04-0.05 | 30 | 32 | 0.94 | 300X |
| PBVI-OG | 3 | 0.05-0.06 | 13 | 13 | 1.00 | 300X |
| PBVI-OG | 3 | 0.0-0.01 | 5 | 13 | 0.38 | 600X |
| PBVI-OG | 3 | 0.01-0.02 | 25 | 31 | 0.81 | 600X |
| PBVI-OG | 3 | 0.02-0.03 | 21 | 22 | 0.95 | 600X |
| PBVI-OG | 3 | 0.03-0.04 | 29 | 29 | 1.00 | 600X |
| PBVI-OG | 3 | 0.04-0.05 | 34 | 34 | 1.00 | 600X |
| PBVI-OG | 3 | 0.05-0.06 | 12 | 12 | 1.00 | 600X |
| PBVI-OG | 3 | 0.0-0.01 | 10 | 13 | 0.77 | 1,200X |
| PBVI-OG | 3 | 0.01-0.02 | 31 | 31 | 1.00 | 1,200X |
| PBVI-OG | 3 | 0.02-0.03 | 22 | 22 | 1.00 | 1,200X |
| PBVI-OG | 3 | 0.03-0.04 | 29 | 29 | 1.00 | 1,200X |
| PBVI-OG | 3 | 0.04-0.05 | 34 | 34 | 1.00 | 1,200X |
| PBVI-OG | 3 | 0.05-0.06 | 12 | 12 | 1.00 | 1,200X |
| VarDict | 1 | 0.0-0.01 | 0 | 12 | 0.00 | 150X |
| VarDict | 1 | 0.01-0.02 | 23 | 27 | 0.85 | 150X |
| VarDict | 1 | 0.02-0.03 | 33 | 33 | 1.00 | 150X |
| VarDict | 1 | 0.03-0.04 | 21 | 21 | 1.00 | 150X |
| VarDict | 1 | 0.04-0.05 | 34 | 34 | 1.00 | 150X |
| VarDict | 1 | 0.05-0.06 | 15 | 15 | 1.00 | 150X |
| VarDict | 1 | 0.0-0.01 | 0 | 9 | 0.00 | 300X |
| VarDict | 1 | 0.01-0.02 | 25 | 30 | 0.83 | 300X |
| VarDict | 1 | 0.02-0.03 | 32 | 32 | 1.00 | 300X |
| VarDict | 1 | 0.03-0.04 | 28 | 28 | 1.00 | 300X |
| VarDict | 1 | 0.04-0.05 | 25 | 25 | 1.00 | 300X |
| VarDict | 1 | 0.05-0.06 | 16 | 16 | 1.00 | 300X |
| VarDict | 1 | 0.0-0.01 | 0 | 14 | 0.00 | 600X |
| VarDict | 1 | 0.01-0.02 | 19 | 23 | 0.83 | 600X |
| VarDict | 1 | 0.02-0.03 | 32 | 32 | 1.00 | 600X |
| VarDict | 1 | 0.03-0.04 | 24 | 24 | 1.00 | 600X |
| VarDict | 1 | 0.04-0.05 | 24 | 24 | 1.00 | 600X |
| VarDict | 1 | 0.05-0.06 | 18 | 18 | 1.00 | 600X |
| VarDict | 1 | 0.0-0.01 | 0 | 14 | 0.00 | 1,200X |
| VarDict | 1 | 0.01-0.02 | 19 | 23 | 0.83 | 1,200X |
| VarDict | 1 | 0.02-0.03 | 32 | 32 | 1.00 | 1,200X |
| VarDict | 1 | 0.03-0.04 | 24 | 24 | 1.00 | 1,200X |
| VarDict | 1 | 0.04-0.05 | 24 | 24 | 1.00 | 1,200X |
| VarDict | 1 | 0.05-0.06 | 18 | 18 | 1.00 | 1,200X |
| VarDict | 2 | 0.0-0.01 | 0 | 19 | 0.00 | 150X |
| VarDict | 2 | 0.01-0.02 | 21 | 26 | 0.81 | 150X |
| VarDict | 2 | 0.02-0.03 | 26 | 27 | 0.96 | 150X |
| VarDict | 2 | 0.03-0.04 | 31 | 32 | 0.97 | 150X |
| VarDict | 2 | 0.04-0.05 | 25 | 25 | 1.00 | 150X |
| VarDict | 2 | 0.05-0.06 | 14 | 14 | 1.00 | 150X |
| VarDict | 2 | 0.0-0.01 | 0 | 15 | 0.00 | 300X |
| VarDict | 2 | 0.01-0.02 | 23 | 28 | 0.82 | 300X |
| VarDict | 2 | 0.02-0.03 | 29 | 29 | 1.00 | 300X |
| VarDict | 2 | 0.03-0.04 | 33 | 33 | 1.00 | 300X |
| VarDict | 2 | 0.04-0.05 | 22 | 22 | 1.00 | 300X |
| VarDict | 2 | 0.05-0.06 | 18 | 18 | 1.00 | 300X |
| VarDict | 2 | 0.0-0.01 | 0 | 13 | 0.00 | 600X |
| VarDict | 2 | 0.01-0.02 | 21 | 29 | 0.72 | 600X |
| VarDict | 2 | 0.02-0.03 | 25 | 25 | 1.00 | 600X |
| VarDict | 2 | 0.03-0.04 | 24 | 24 | 1.00 | 600X |
| VarDict | 2 | 0.04-0.05 | 29 | 29 | 1.00 | 600X |
| VarDict | 2 | 0.05-0.06 | 16 | 16 | 1.00 | 600X |
| VarDict | 2 | 0.0-0.01 | 0 | 13 | 0.00 | 1,200X |
| VarDict | 2 | 0.01-0.02 | 21 | 29 | 0.72 | 1,200X |
| VarDict | 2 | 0.02-0.03 | 25 | 25 | 1.00 | 1,200X |
| VarDict | 2 | 0.03-0.04 | 24 | 24 | 1.00 | 1,200X |
| VarDict | 2 | 0.04-0.05 | 29 | 29 | 1.00 | 1,200X |
| VarDict | 2 | 0.05-0.06 | 16 | 16 | 1.00 | 1,200X |
| VarDict | 3 | 0.0-0.01 | 0 | 18 | 0.00 | 150X |
| VarDict | 3 | 0.01-0.02 | 24 | 28 | 0.86 | 150X |
| VarDict | 3 | 0.02-0.03 | 20 | 22 | 0.91 | 150X |
| VarDict | 3 | 0.03-0.04 | 34 | 34 | 1.00 | 150X |
| VarDict | 3 | 0.04-0.05 | 32 | 32 | 1.00 | 150X |
| VarDict | 3 | 0.05-0.06 | 13 | 13 | 1.00 | 150X |
| VarDict | 3 | 0.0-0.01 | 0 | 13 | 0.00 | 300X |
| VarDict | 3 | 0.01-0.02 | 25 | 32 | 0.78 | 300X |
| VarDict | 3 | 0.02-0.03 | 27 | 28 | 0.96 | 300X |
| VarDict | 3 | 0.03-0.04 | 29 | 29 | 1.00 | 300X |
| VarDict | 3 | 0.04-0.05 | 32 | 32 | 1.00 | 300X |
| VarDict | 3 | 0.05-0.06 | 13 | 13 | 1.00 | 300X |
| VarDict | 3 | 0.0-0.01 | 0 | 13 | 0.00 | 600X |
| VarDict | 3 | 0.01-0.02 | 21 | 31 | 0.68 | 600X |
| VarDict | 3 | 0.02-0.03 | 21 | 22 | 0.95 | 600X |
| VarDict | 3 | 0.03-0.04 | 29 | 29 | 1.00 | 600X |
| VarDict | 3 | 0.04-0.05 | 34 | 34 | 1.00 | 600X |
| VarDict | 3 | 0.05-0.06 | 12 | 12 | 1.00 | 600X |
| VarDict | 3 | 0.0-0.01 | 0 | 13 | 0.00 | 1,200X |
| VarDict | 3 | 0.01-0.02 | 21 | 31 | 0.68 | 1,200X |
| VarDict | 3 | 0.02-0.03 | 21 | 22 | 0.95 | 1,200X |
| VarDict | 3 | 0.03-0.04 | 29 | 29 | 1.00 | 1,200X |
| VarDict | 3 | 0.04-0.05 | 34 | 34 | 1.00 | 1,200X |
| VarDict | 3 | 0.05-0.06 | 12 | 12 | 1.00 | 1,200X |

Supplementary Table 3. Mean minimum VAF detected for every combination of reference allele and alternate nucleotide in *PIK3CA* at 600X test sample depth.

|  | Alternate nucleotide | | | |
| --- | --- | --- | --- | --- |
| Reference allele | A | C | G | T |
| A | - | 0.01178 | 0.01140 | 0.01125 |
| C | 0.01349 | - | 0.01115 | 0.01214 |
| G | 0.01226 | 0.01107 | - | 0.01291 |
| T | 0.01126 | 0.01148 | 0.01184 | - |

Supplementary Table 4. Number of variants identified by PBVI-OG, LoFreq, and Vardict variant callers.

| Sample ID | Number of Identified Variants* | | | Average DOC |
| --- | --- | --- | --- | --- |
|  | PBVI | LoFreq | VarDict |  |
| PS105.3_TL | 2 | 0 | 55 | 183 |
| PS105.3_TR | 2 | 0 | 61 | 161 |
| PS137.3_1577 | 3 | 1 | 88 | 165 |
| PS235.3_248 | 2 | 1 | 61 | 174 |
| PS118.3 | 8 | 2 | 61 | 168 |
| PS149.3_spleen | 3 | 0 | 19 | 233 |
| PS279.3_RF | 8 | 1 | 11 | 359 |
| PS286.3_T | 2 | 1 | 30 | 212 |
| PS299.3 | 13 | 1 | 49 | 202 |
| PS305.3_SF1 | 7 | 5 | 30 | 244 |
| PS312.3_SF1 | 1 | 1 | 43 | 185 |
| PS313.3_T | 2 | 1 | 32 | 192 |
| PS317.3_SF2(2) | 8 | 2 | 4 | 450 |
| PS322.3 | 11 | 1 | 29 | 225 |
| PS323.3 | 8 | 0 | 29 | 232 |
| PS326.3 | 4 | 1 | 62 | 157 |
| PS328.3_T | 5 | 0 | 6 | 275 |
| PS331.3_2340 | 11 | 1 | 1 | 683 |
| PS333.3 | 10 | 1 | 55 | 192 |
| PS337.3_LT | 3 | 0 | 106 | 185 |
| PS338.3_176 | 9 | 0 | 2 | 455 |
| PS340.3_378 | 3 | 0 | 6 | 328 |
| PS341.3_409 | 5 | 0 | 17 | 247 |
| PS342.3_556 | 11 | 0 | 15 | 253 |
| PS343.3_538 | 10 | 0 | 6 | 381 |
| PS344.3_844 | 12 | 0 | 10 | 346 |
| PS346.3_SF1 | 8 | 2 | 19 | 270 |

*Counts include single nucleotide variants filtered for <10 alleles in gnomAD. DOC, Depth of coverage.

Supplementary Table 5.Variants of interest identified in 27 overgrowth samples.

| Sample ID | Variants of Interest | | | VAF | Depth | Alt Count | PBVI  Strand -Aware  P-value | Number of Variants Called | | | Confirmed | Pathogenicity | COSMIC v92  (mutation count) | ClinVar  Variant ID | Overgrowth |
| --- | --- | --- | --- | --- | --- | --- | --- | --- | --- | --- | --- | --- | --- | --- | --- |
|  | cDNA Nomenclature | Gene | Protein Nomenclature |  |  |  |  | PBVI | LoFreq | Vardict |  |  |  |  |  |
| PS105.3_TL | NM_001014431.1:c.49G>A | *AKT1* | p.Glu17Lys | 0.026 | 192 | 5 | 3.28E-03 | Yes | No | Yes | Restriction Digest | Pathogenic | COSV62571334  (767) | 13983 | PMID: 21793738 |
| PS105.3_TR | NM_001014431.1:c.49G>A | *AKT1* | p.Glu17Lys | 0.028 | 176 | 5 | 1.227E-02 | Yes | No | Yes | Restriction Digest | Pathogenic | COSV62571334  (767) | 13983 | PMID: 21793738 |
| PS137.3_1577 | NM_006218.3:c.1636C>A | *PIK3CA* | p.Gln546Lys | 0.065 | 247 | 16 | 1.027E-16 | Yes | Yes | Yes | NA | Likely Pathogenic | COSV55873527  (254) | 13657 | PMID: 28151489 |
| PS235.3_248 | NM_006218.3:c.1633G>A | *PIK3CA* | p.Glu545Lys | 0.027 | 256 | 7 | 1.433E-05 | Yes | Yes | Yes | ddPCR | Pathogenic | COSV55873239  (2,918) | 13655 | PMID: 22729223; PMID: 22729224 |
| PS118.3 | NM_001014431.1:c.49G>A | *AKT1* | p.Glu17Lys | 0.272 | 169 | 46 | 1.265E-93 | Yes | Yes | Yes | Restriction Digest | Pathogenic | COSV62571334  (767) | 13983 | PMID: 21793738 |
| PS149.3_spl | NM_006218.3:c.1633G>A | *PIK3CA* | p.Glu545Lys | 0.012 | 515 | 6 | 2.520E-02 | Yes | No | Yes | ddPCR | Pathogenic | COSV55873239  (2,918) | 13655 | PMID: 22729223; PMID: 22729224 |
| PS279.3_RF | NM_006218.3:c.241G>A | *PIK3CA* | p.Glu81Lys | 0.372 | 739 | 275 | 0.000E+00 | Yes | Yes | Yes | Sanger Sequencing | Pathogenic | COSV55873676  (97) | 376478 | PMID: 22729224 |
| PS286.3_T | NM_006218.3:c.1625A>T | *PIK3CA* | p.Glu542Val | 0.314 | 379 | 119 | 6.877E-177 | Yes | Yes | Yes | Sanger Sequencing | Likely Pathogenic | COSV55881194  (20) | 376474 | PMID: 26627007 |
| PS299.3 | NM_006218.3:c.3140A>G | *PIK3CA* | p.His1047Arg | 0.045 | 356 | 16 | 6.138E-19 | Yes | No | Yes | ddPCR | Pathogenic | COSV55873195  (3,656) | 13652 | PMID: 22658544; PMID: 22729222 |
| PS305.3_SF1 | NM_181523.2:c.1690A>G | *PIK3R1* | p.Asn564Asp | 0.243 | 321 | 78 | 2.066E-142 | Yes | Yes | Yes | NA | VUS | COSV57124003  (48) | 376261 |  |
| PS312.3_SF1 | NM_006218.3:c.1412C>T | *PIK3CA* | p.Pro471Leu | 0.082 | 341 | 28 | 2.181E-33 | Yes | Yes | Yes | NA | Likely Pathogenic | COSV55911123  (15) | 376359 | PMID: 28151489 |
| PS313.3_T | NM_006218.3:c.3140A>G | *PIK3CA* | p.His1047Arg | 0.307 | 358 | 110 | 2.103E-193 | Yes | Yes | Yes | Sanger Sequencing | Pathogenic | COSV55873195  (3,656) | 13652 | PMID: 22658544; PMID: 22729222 |
| PS317.3_SF2 | NM_001014431.1:c.49G>A | *AKT1* | p.Glu17Lys | 0.077 | 491 | 38 | 4.058E-56 | Yes | Yes | Yes | Restriction Digest | Pathogenic | COSV62571334  (767) | 13983 | PMID: 21793738 |
| PS322.3 | NM_006218.3:c.1637A>G | *PIK3CA* | p.Gln546Arg | 0.473 | 300 | 142 | 2.015E-237 | Yes | Yes | Yes | Sanger Sequencing | Pathogenic | COSV55876869  (117) | 45466 | PMID: 28151489; PMID: 29174369 |
| PS322.3 | NM_194449.3:c.873C>G | *PHLPP1* | p.Phe291Leu | 0.018 | 396 | 7 | 1.926E-02 | Yes | No | No | NA | VUS | COSV53026219  (1) | NA |  |
| PS323.3 | NM_006218.3:c.1132T>C | *PIK3CA* | p.Cys378Arg | 0.029 | 240 | 7 | 1.562E-04 | Yes | No | Yes | NA | Likely Pathogenic | COSV55882697  (14) | 917489 | PMID: 28502725 |
| PS326.3 | NM_006218.3:c.1258T>C | *PIK3CA* | p.Cys420Arg | 0.153 | 189 | 29 | 3.549E-43 | Yes | Yes | Yes | Sanger Sequencing | Pathogenic | COSV55874020  (203) | 31945 | PMID: 22658544 |
| PS328.3_T | NM_002072.4:c.317A>T | *GNAQ* | p.Asn106Ile | 0.017 | 344 | 6 | 9.847E-04 | Yes | No | No | NA | VUS | COSV54109153  (4) | NA |  |
| PS333.3 | NM_001014431.1:c.49G>A | *AKT1* | p.Glu17Lys | 0.194 | 191 | 37 | 3.000E-70 | Yes | Yes | Yes | Restriction Digest | Pathogenic | COSV62571334  (767) | 13983 | PMID: 21793738 |
| PS341.3_409 | NM_006218.3:c.1633G>A | *PIK3CA* | p.Glu545Lys | 0.018 | 455 | 8 | 3.621E-05 | Yes | No | Yes | ddPCR | Pathogenic | COSV55873239  (2,918) | 13655 | PMID: 22729223 ; PMID: 22729224 |
| PS341.3_409 | NM_033360.3:c.178G>A | *KRAS* | p.Gly60Ser | 0.020 | 256 | 5 | 2.059E-02 | Yes | No | Yes | NA | VUS | NA | 12597 | PMID: 19396835 |
| PS342.3_556 | NM_006218.3:c.213A>G | *PIK3CA* | p.Val71= | 0.013 | 470 | 6 | 8.870E-04 | Yes | No | Yes | NA | VUS | COSV55880259  (2) | 246683 |  |
| PS344.3_844* | NM_006218.3:c.1633G>A | *PIK3CA* | p.Glu545Lys | 0.008 | 615 | 5 | 1.250E+00 | No | No | No | ddPCR | Pathogenic | COSV55873239  (2,918) | 13655 | PMID: 22729223; PMID: 22729224 |
| PS344.3_844 | NM_181523.2:c.2047G>A | *PIK3R1* | p.Glu683Lys | 0.013 | 779 | 10 | 1.533E-05 | Yes | No | Yes | NA | VUS | COSV57122880  (4) | NA |  |
| PS344.3_844 | NM_002072.4:c.95A>G | *GNAQ* | p.Asp32Gly | 0.013 | 742 | 10 | 2.245E-02 | Yes | No | No | NA | VUS | COSV54128556  (1) | NA |  |
| PS346.3_SF1 | NM_006218.3:c.1624G>A | *PIK3CA* | p.Glu542Lys | 0.038 | 476 | 18 | 2.040E-16 | Yes | Yes | Yes | ddPCR | Pathogenic | COSV55873227  (1,814) | 31944 | PMID: 22658544 |

*This variant was not called by the OverGrowth Position-Based model and was identified by manual inspection of the data.
